# The CD160–HVEM axis at the crossroads of tolerance and immunity: lessons from cancer for autoimmunity and transplantation

**DOI:** 10.3389/fimmu.2026.1782124

**Published:** 2026-10-06

**Authors:** Shulin Sun, Lulin Ma

**Affiliations:** Department of Urology, Peking University Third Hospital, Beijing, China

**Keywords:** autoimmunity, BTLA, CD160–HVEM axis, clinical translation, NK cell, T cell exhaustion, transplantation

## Abstract

**Background:**

The clinical success of immune checkpoint blockade (ICB) in oncology has validated the existence of molecular brakes that restrain T-cell immunity. While releasing these brakes unleashes antitumour responses, their physiological role is to maintain self-tolerance. This review explores the reciprocal paradigm of the CD160–HVEM axis across three disease settings: immuno-oncology, autoimmunity and transplantation. CD160–HVEM signalling can enforce pathological T-cell exhaustion in cancer, yet its failure or costimulatory co-option can precipitate autoimmunity or accelerate allograft rejection.

**Mechanisms:**

Unlike programmed cell death protein 1 (PD-1), CD160 operates within a bidirectional network involving herpesvirus entry mediator (HVEM; TNFRSF14), B- and T-lymphocyte attenuator (BTLA) and LIGHT (TNFSF14). The axis is expressed across lymphocytes, myeloid cells, epithelium, endothelium and malignant B cells. Mouse and human biology of the axis are not interchangeable: murine CD160 is a broad major histocompatibility complex (MHC) class I and CD1d receptor, whereas human natural killer (NK) triggering is dominated by HLA-C, and the transmembrane isoform CD160-TM is human-characterised. Structural diversity, from an inhibitory glycosylphosphatidylinositol (GPI)-anchored form to activating CD160-TM on NK cells, positions CD160 as a tunable rheostat rather than a linear checkpoint.

**Therapeutic implications:**

While direct anti-CD160 candidates remain in advanced preclinical development, other components of the HVEM network are undergoing human clinical testing. The most mature clinical programme is tifcemalimab, an anti-BTLA monoclonal antibody, not an anti-CD160 agent. Antagonistic anti-HVEM antibodies (Demerlé anti-HVEM18–10 in humanised mice; preclinical fully human 4CB1, mechanistically related but distinct) block HVEM engagement by both BTLA and CD160. Because CD160 is inhibitory on T cells but activating on NK cells via CD160-TM, a systemic antibody to the shared ectodomain is a dual-function drug-design constraint, not a simple checkpoint blockade.

**Conclusion:**

Precision use of this axis requires isoform, cell type, interaction geometry and disease context to be specified before a direction of intervention is chosen. Next steps are biomarker-qualified enrichment, isoform-discriminating reagents, and correlative CD160 profiling inside ongoing BTLA–HVEM studies. Pathological exhaustion that shields tumours remains a map for restoring tolerance in autoimmunity and for blocking CD28-independent rejection in transplantation.

## 1 Introduction: cellular geography of the CD160–HVEM axis

### 1.1 Checkpoints, tolerance, and the reciprocal paradigm

The advent of ICB has fundamentally reshaped the landscape of cancer therapy, validating the concept that the immune system is not merely passive but actively restrained by specific molecular brakes (1). The clinical success of monoclonal antibodies targeting PD-1/programmed death-ligand 1 (PD-L1) and cytotoxic T-lymphocyte-associated protein 4 (CTLA-4) has demonstrated that releasing these brakes can unleash potent antitumour immunity, leading to durable responses in malignancies previously considered untreatable (2). However, this therapeutic triumph has also illuminated a critical biological corollary: these same checkpoint pathways are the physiological guardians of immune tolerance, preventing the immune system from attacking self-tissues (3).

This duality presents a reciprocal paradigm for translational immunology. Just as cancer immunotherapy seeks to break tolerance to eradicate tumours, the treatment of autoimmunity and transplantation requires the restoration or induction of tolerance to preserve tissue integrity (2). The mechanisms of resistance to current ICB therapies—where tumours exploit alternative or compensatory inhibitory pathways to maintain an immunosuppressive microenvironment—offer a roadmap for identifying novel tolerance-inducing targets. In this context, exhausted T cells in cancer, characterised by the co-expression of multiple inhibitory receptors, represent a state of deep tolerance that autoimmune therapies aspire to mimic (4).

Despite the transformative impact of PD-1/PD-L1 inhibitors, a significant proportion of patients fail to respond (primary resistance) or relapse after an initial response (acquired resistance) (5). In colorectal cancer (CRC), for instance, the efficacy of anti-PD-1 therapy is largely restricted to tumours with high microsatellite instability (MSI-H), while the vast majority of microsatellite-stable (MSS) tumours remain refractory (6). This clinical reality underscores a fundamental limitation: targeting the PD-1 axis alone is often insufficient to fully reinvigorate the immune response, particularly in the context of deep T-cell exhaustion.

Recent high-resolution profiling of the tumour microenvironment (TME) has revealed that T-cell exhaustion is not a monolithic state but a progressive differentiation trajectory. While PD-1 blockade can revitalise progenitor-exhausted T cells, it is less effective against terminally exhausted subsets, which co-express multiple inhibitory receptors and exhibit epigenetic stability (7).

### 1.2 The molecules in brief

To transcend the limitations of current checkpoint therapies, we must move beyond the binary ligand–receptor paradigm exemplified by PD-1/PD-L1. The CD160–HVEM axis emerges as a critical frontier in this regard. Unlike the linear PD-1 pathway, CD160 functions as a central node within a multipartite signalling network that includes HVEM, BTLA and LIGHT (8, 9). HVEM functions as a molecular switchboard rather than a simple linear receptor: LIGHT delivers costimulatory signals, whereas BTLA and CD160 transmit inhibitory signals into HVEM-bearing T cells (10, 11). CD160 itself exhibits remarkable structural plasticity; it exists as both an inhibitory GPI-anchored protein and an activating transmembrane receptor (CD160-TM) (12, 13). This duality positions CD160 not merely as a redundant checkpoint, but as a tunable rheostat of immune function—capable of enforcing exhaustion programmes in T cells or triggering cytotoxicity in NK cells depending on the isoform and cellular context (12, 14). Binding kinetics, cis versus trans geometry, and isoform biochemistry are developed in Section 2. First, however, the axis must be placed on the cells that actually carry it.

### 1.3 Where the axis operates: CD160- and HVEM-expressing cells

The CD160–HVEM axis is not a T-cell checkpoint that happens to appear elsewhere. It is a broadly distributed receptor–ligand system whose output is set by which cell carries which partner. A systemic agent will therefore engage every pairing listed below, not only the pairing that motivated the indication.

CD160-expressing cells. On circulating cytotoxic NK cells (CD56dim CD16^+^), CD160 is an activating receptor: engagement by HLA-C constitutively expressed on K562 cells triggers cytotoxicity, and HLA-C is the physiological MHC class I ligand for human NK CD160 (15, 16). Upon NK activation, alternative splicing induces CD160-TM, which recruits p56lck and amplifies extracellular signal-regulated kinase (ERK) 1/2-dependent effector function (12). HVEM costimulates CD160 on effector NK cells—enhancing interferon-γ (IFN-γ), tumour necrosis factor-α (TNF-α) and cytolysis via ERK1/2 and Akt (protein kinase B)—whereas HVEM engagement of BTLA on the same lineage is inhibitory (17).

On T cells the picture splits by lineage. Human CD4^+^ T cells receive an inhibitory signal when CD160 ligates HVEM, reducing activation and proliferation (18). A minor CD4^+^CD160^+^ subset infiltrates inflamed skin and can co-express the NK receptor CD158k in normal skin and mycosis fungoides (19, 20). On circulating CD28-negative cytotoxic effector CD8^+^ T cells, GPI-anchored CD160 can instead act as a T-cell receptor (TCR) co-receptor that couples to p56lck and phosphorylated CD3ζ, partially compensating for absent CD28 costimulation (21). In CRC, CD160^+^CD8^+^ T cells annotate a stem-like, T cell factor 1 (Tcf1; encoded by *TCF7*)^+^ progenitor-exhausted pool whose maintenance, not its blockade, is linked to PD-1 responsiveness (14).

IELs were essentially absent from the previous version of this review, yet they are a principal *in vivo* setting in which CD160 on lymphocytes engages HVEM on epithelium. CD160^+^ IELs promote intestinal regeneration after chemotherapy through CD160/HVEM signalling, and CD160^+^ IELs together with CCRL2^+^ macrophages have been implicated in differential repair after cardiac and liver injury (22, 23). Mast cells constitutively express and secrete CD160-GPI, supplying local soluble CD160 capable of engaging HVEM on CD8^+^ T cells (24). Endothelial cells express CD160; soluble HLA-G1 binds this receptor and triggers apoptosis, thereby inhibiting angiogenesis (25). Finally, CD160 is a tumour-specific B-lineage antigen: it is detected in 98% of chronic lymphocytic leukaemia (CLL) and 100% of hairy-cell leukaemia (HCL) cases, is uncommon in other B-cell lymphoproliferative disorders, and is absent from the normal B-cell hierarchy (26). In CLL, CD160 signalling mediates phosphoinositide 3-kinase (PI3K)-dependent survival (27). CD160 on malignant B cells is therefore a tumour-intrinsic target, not a surrogate for T-cell CD160.

HVEM-expressing cells. HVEM on T cells is the canonical switchboard described above (9, 28). Constitutive HVEM expression on human B cells is documented by immunohistochemistry on mantle-zone and marginal-zone B cells and by flow cytometry showing that circulating B cells co-express HVEM and BTLA (29, 30). NK cells express HVEM as well as CD160; ligand choice (CD160 versus BTLA) sets valence (17). DCs participate in the HVEM network more clearly through BTLA than through CD160: in urothelial cancer, circulating and tumour CD1c^+^, CD141^+^ and plasmacytoid DCs expressed BTLA and T cell immunoglobulin and mucin domain-containing protein 3 (TIM-3) but not CD160, PD-1 or CTLA-4 (31). HVEM protein has been reported on immature and LPS-activated human dendritic cells in a peripheral-blood panel; a dedicated tumour-DC HVEM atlas is still lacking (30). Circulating monocytes can carry high HVEM; in pancreatic ductal adenocarcinoma (PDAC), increased monocyte HVEM predicted poorer survival and compared favourably with CA19–9 as a prognostic marker in that cohort (32). Epithelial HVEM is the counter-receptor for IEL CD160 in the intestine (22). HVEM on mesenchymal or stromal cells was named by Reviewer 5; we did not locate a primary source in this repository and therefore do not assert expression. Any such pairing remains hypothesis-generating.

Design implication. Because CD160 and HVEM are distributed across lymphocytes, myeloid cells, epithelium, endothelium and, in CLL/HCL, the malignant clone itself, a systemic mAb, fusion protein or peptide will simultaneously engage pairs with opposite valences. Cell geography is therefore not background: it is the first constraint on drug design (Sections 3.4 and 6).

### 1.4 Mouse versus human biology of the axis

Conclusions transfer imperfectly between species, and the manuscript’s previous mouse-versus-human content was a single clause. Murine CD160 is an immunoglobulin-like receptor on NK cells, NKT cells and activated CD8^+^ T cells that recognises classical and non-classical MHC class I molecules, including CD1d, and regulates NK activation (33). In the mouse, CD160 also marks CD44hi memory CD8^+^ T cells (34). Human NK cells, by contrast, use HLA-C as the physiological MHC ligand for CD160 (15). Soluble HLA-G1 is an endothelial CD160 ligand in humans (25); mice lack a true HLA-G orthologue, so this vascular pairing does not have a one-to-one murine counterpart. Mouse liver type 1 innate lymphoid cells (ILC1) can be split by granzyme A and CD160, a partition without a clean human equivalent in the papers reviewed here (35). Mast-cell CD160-GPI is conserved in human and mouse (24).

CD160-TM was identified and functionally characterised in human NK cells (12). We do not assume a one-to-one murine transmembrane story. Binding of HVEM by CD160-TM is detectable but less pronounced than binding by CD160-GPI in human T-cell assays (36). *In vivo*, HVEM muteins show that the LIGHT arm and the immunoglobulin-ligand (BTLA/CD160) arm are separable: LIGHT-selective function contributes to intestinal bacterial clearance, whereas immunoglobulin-ligand function ameliorates liver inflammation (37). Testing an anti-human HVEM antibody required a humanised HVEM/BTLA double knock-in (38). Most transplantation *in vivo* evidence for CD160 is murine (Section 5). These asymmetries are why we grade claims by species as well as by experimental system.

### 1.5 How evidence is graded

Throughout, we grade the strength of evidence supporting each translational claim using four descriptors: “established” (supported by multiple independent studies), “strong preclinical” (reproducible in animal models and/or human tissue with mechanistic support), “limited correlative human” (human expression or genetic association without established causality), and “hypothesis-generating” (single studies, *in vitro* data, or conceptual extrapolation).

### 1.6 Scope and structure of this review

We then treat immuno-oncology, autoimmunity and transplantation in turn, addressing in each the key genetic link, the most relevant cell types, the *in vivo* models, and the translational and clinical data (efficacy, safety, patient population, and line of therapy). Vascular and angio-immune biology is retained as a named subsection of immuno-oncology, where its strongest *in vivo* evidence belongs, rather than as a fourth disease chapter. While direct anti-CD160 candidates remain in advanced preclinical development, other components of the HVEM network are actively undergoing human clinical testing. Infection-model material is compressed to the minimum required to explain why CD160 can be inhibitory or activating (Section 2.3); we do not review viral immunology as a disease area.

## 2 Molecular logic of the HVEM switchboard

### 2.1 Binding, cis versus trans, and the interaction matrix

Tolerance is maintained by HVEM (TNFRSF14), which functions as a molecular switchboard rather than a simple linear receptor (11) (**Figure 1**). HVEM integrates a diverse repertoire of ligands with opposing functions: it binds the TNF superfamily member LIGHT (TNFSF14) to deliver costimulatory signals, while its interaction with immunoglobulin superfamily members—specifically BTLA and CD160—transmits inhibitory signals (9, 10). This capacity to integrate conflicting inputs places the HVEM network at the fulcrum of immune decision-making.

**Figure 1:**
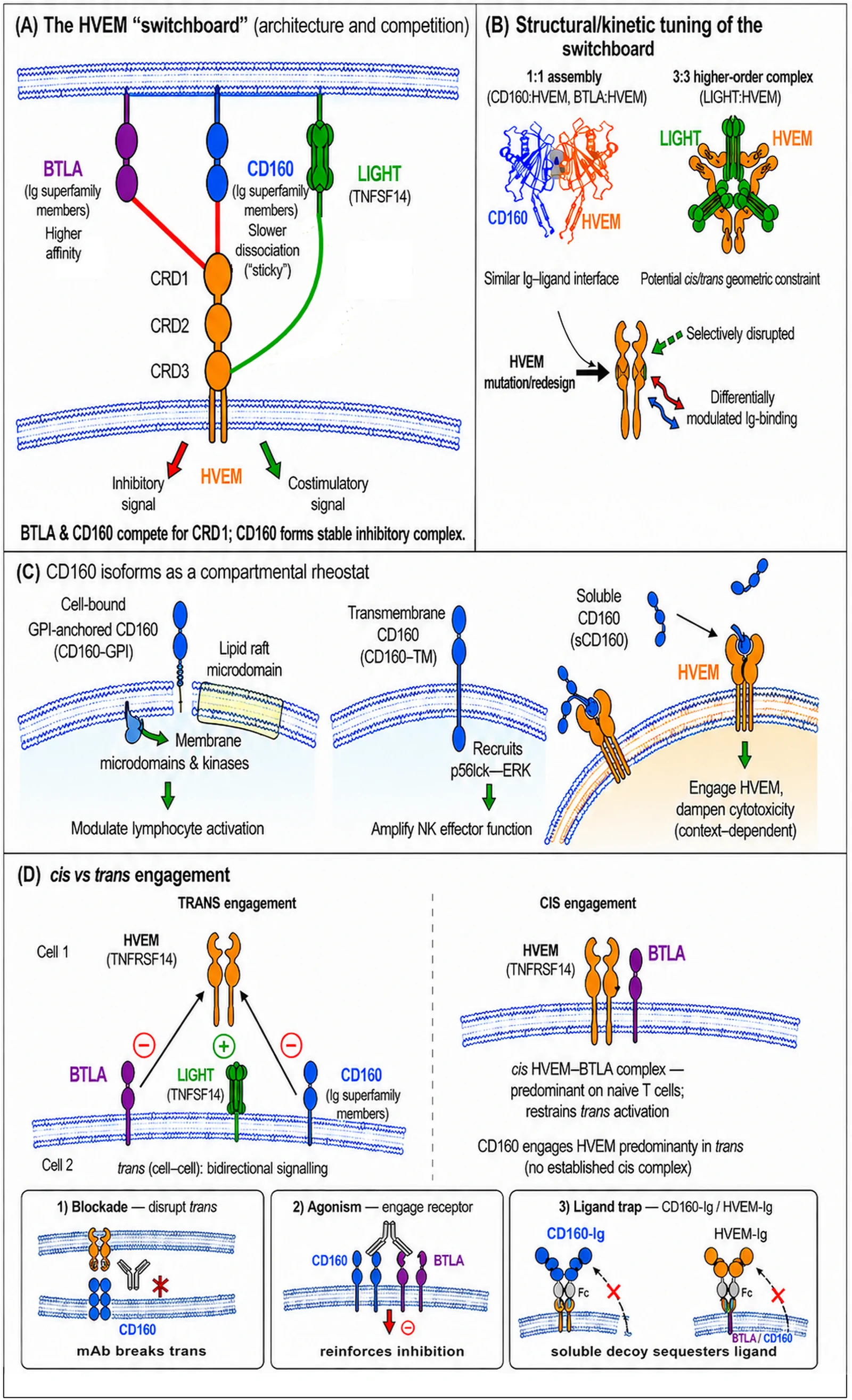
The CD160–HVEM signalling network and structural diversity. **(A)** HVEM “switchboard”: HVEM (TNFRSF14) integrates opposing inputs: LIGHT (TNFSF14) binds CRD2/CRD3 (costimulation), whereas BTLA and CD160 bind CRD1 (inhibition). BTLA binds with higher affinity, while CD160 has a slower off-rate, consistent with more durable inhibitory occupancy. **(B)** Structural tuning: CD160:HVEM forms a 1:1 complex with a BTLA-like interface, whereas LIGHT engages HVEM in a 3:3 assembly (potential cis/trans constraints). HVEM point mutations and interface redesign can bias ligand selectivity to functionally split competing pathways. **(C)** CD160 isoform rheostat (by compartment): CD160-GPI signals via membrane microdomains/associated kinases; CD160-TM signals through an intracellular tail (e.g., p56lck→ERK) to amplify NK effector function; soluble CD160 can act as a circulating HVEM ligand (“soluble checkpoint”) in a context- and format-dependent manner. Evidence levels: the HVEM–ligand competition and isoform signalling **(A, C)** are established; the structural cis/trans inferences and ligand-selectivity engineering **(B)** are strong preclinical (structural and *in vitro* data). **(D)** cis versus trans engagement: In trans (between apposing cells), HVEM on one cell is engaged by CD160 and BTLA (both inhibitory, red) or by LIGHT (costimulatory, green) on the other, producing bidirectional signalling. In cis (same membrane), HVEM and BTLA form a heterodimer that predominates on naïve T cells and restrains trans activation, whereas CD160 engages HVEM predominantly in trans (no established cis complex). These configurations frame three therapeutic modalities (bottom): blockade (anti-HVEM/anti-CD160 mAb) disrupts the trans interaction; agonism (agonist mAb or HVEM-Ig) clusters the inhibitory receptor to reinforce inhibition; and ligand traps (soluble CD160-Ig or HVEM-Ig) sequester the ligand to prevent engagement.

Within this competitive landscape, CD160 acts as a critical counterbalance to LIGHT. Mechanistically, while LIGHT binds to cysteine-rich domain (CRD) 2 and CRD3 of HVEM to drive T-cell proliferation, CD160 engages CRD1, antagonising this costimulatory activity (9). Recent structural insights have revealed a key distinction between the inhibitory ligands: although BTLA exhibits higher binding affinity for HVEM, CD160 displays a significantly slower dissociation rate (39). This kinetic property suggests that CD160 forms a highly stable complex with HVEM, enforcing a more durable inhibitory signal compared with the more transient interactions of BTLA.

This competitive architecture is supported by structure-guided work: crystal structures of CD160 and the CD160:HVEM complex indicate a 1:1 assembly and an interface similar to BTLA: HVEM, consistent with overlapping binding sites for the two immunoglobulin ligands (40). In contrast, LIGHT engages HVEM in a higher-order 3:3 assembly, which may impose geometric constraints relevant to cis versus trans engagement. Importantly, HVEM appears to be tunable rather than fixed: a point mutation (G89F) can directly disrupt LIGHT binding yet differentially modulate immunoglobulin-ligand binding, enhancing CD160 binding while decreasing BTLA binding in cell-based assays (41). Complementing this mutation-as-probe approach, computational interface redesign (ProtLID) produced HVEM single- or double-mutant variants with selective binding to only one or two of the three cognate ligands (LIGHT, BTLA, CD160), providing tools to functionally decouple the switchboard (42). Finally, hydrogen–deuterium exchange mass spectrometry (HDX-MS) coupled with molecular modelling has been used to map CD160 fragments engaging HVEM and to derive a predictive three-dimensional model of the CD160–HVEM complex to inform inhibitor design (43).

Furthermore, this regulation is bidirectional. Beyond receiving signals (reverse signalling), CD160 can act as a ligand to transmit inhibitory signals into HVEM-expressing cells (forward signalling). For instance, CD160 ligation of HVEM on CD4^+^ T cells directly inhibits their activation and proliferation, providing a mechanism for T cell-mediated suppression (18).

A further layer of regulation derives from the spatial orientation of these interactions. HVEM can engage its ligands either in trans, between two apposing cells across the immunological synapse, or in cis, with a ligand co-expressed in the same membrane. The best-characterised cis interaction is the HVEM–BTLA heterodimer, which is the predominant configuration on naïve T cells and functions as an intrinsic brake: by occupying HVEM, the cis HVEM–BTLA complex prevents HVEM from being activated in trans by ligands in the surrounding microenvironment, attenuating nuclear factor-κB (NF-κB)–dependent survival signalling and helping maintain the naïve state (28, 44). CD160, by contrast, has not been shown to form an equivalent cis complex; it acts predominantly as a trans ligand, and the geometric differences between the 1:1 CD160:HVEM and 3:3 LIGHT: HVEM assemblies (40) likely constrain which engagements are favoured in each configuration. This distinction is therapeutically consequential: agents that disrupt trans engagement (blocking antibodies) differ in effect from agents that engage receptors directly (agonists) or that sequester ligands (ligand traps such as CD160-Ig or HVEM-Ig). For example, an antibody that displaces the cis HVEM–BTLA complex can paradoxically license trans HVEM activation, whereas a cis-stabilising agonist can reinforce inhibition (45). Predicting the outcome of any CD160/HVEM-directed intervention therefore requires specifying not only the molecular form and compartment, but also the cis versus trans geometry of the targeted interaction (**Table 1**).

**Table 1:** CD160/HVEM/BTLA/LIGHT interactions by context and directionality.

Ligand–receptor	Cell/context	cis/trans	Direction	Evidence grade	Refs
LIGHT (TNFSF14)–HVEM	T cells/antigen-presenting cells (APCs)	trans (3:3 assembly)	Costimulatory	established	(9, 10)
BTLA–HVEM	T cells (naïve)	cis (predominant) + trans	Inhibitory (cis complex restrains trans activation)	established	(11, 28, 44)
CD160–HVEM	NK; CD4^+^/CD8^+^ T cells	trans (no established cis; cis is BTLA-specific)	Context-dependent: CD4^+^ T inhibitory/NK costimulatory	established	(9, 17, 18)
CD160–HLA-C	NK	trans	Activating (cytotoxicity)	established	(15)
CD160–MHC-I/CD1d	NK, NKT (murine)	trans	Activation-tuning	strong preclinical	(33)
CD160–soluble HLA-G1	Endothelium	trans (soluble ligand)	Inhibitory/pro-apoptotic (anti-angiogenic)	strong preclinical	(25)
sCD160–HVEM	NK (tumour microenvironment)	soluble	Inhibitory (“soluble checkpoint”)	limited correlative human	(46, 47)
CD160–HVEM (IEL–epithelium)	IELs/intestinal epithelium	trans	Epithelial regeneration (NF-κB)	strong preclinical	(22)
CD160 (malignant B)–HVEM/PI3K	CLL/HCL B cells (absent on normal B cells)	cell-intrinsic	Survival/growth	limited correlative human (expression); strong preclinical (signalling)	(26, 27)
HVEM (monocyte)–CD160/BTLA	Circulating monocytes (PDAC)	not resolved	Associated with poor survival	limited correlative human	(32)

cis, co-expressed on the same cell membrane; trans, engagement between apposed cells. The cis HVEM–BTLA complex is well documented; no equivalent cis CD160–HVEM complex has been established. Species: HLA-C and HLA-G1 rows are human; MHC-I/CD1d is murine.

### 2.2 Isoforms and molecular formats

A defining feature of CD160 biology is its structural heterogeneity, which dictates its functional outcome (12, 13). The predominant isoform on circulating NK and T cells is the GPI-anchored form (CD160-GPI). Lacking an intracellular domain, this isoform relies on lateral associations within the membrane to transduce signals. In NK cells, CD160 mobilises into lipid rafts upon activation, where it physically associates with the co-activating receptor CD2 (48). This clustering facilitates the recruitment of key signalling effectors. Rabot et al. demonstrated that CD160-mediated effector functions—including the release of IFN-γ, TNF-α and interleukin-6 (IL-6)—are strictly dependent on the recruitment of PI3K and the subsequent activation of Akt and ERK, with spleen tyrosine kinase (Syk) acting as a critical upstream initiator in NK cells (49).

Importantly, isoform-specific biology is also evident in T cells. In primary human CD4^+^ and CD8^+^ T cells, both CD160-GPI and CD160-TM transcripts/proteins have been detected, but HVEM binding appears less pronounced for CD160-TM; mechanistically, triggering CD160-GPI (but not CD160-TM) enhanced T-cell activation, and CD160-GPI-specific antibodies synergised with PD-1 blockade to enhance antigen-specific CD8^+^ T cell proliferation ex vivo (36).

However, this membrane-bound form is unstable. Upon activation (for example by IL-15), NK cells release a soluble form of CD160 (sCD160) through metalloprotease-mediated proteolytic cleavage (46). While physiologically functioning to limit tissue damage, this mechanism is actively exploited in cancer. In melanoma, high levels of sCD160 are detected in patient serum and correlate with metastatic progression (>3 sites) (47). Functional studies reveal that this tumour-associated sCD160 binds to HVEM on NK cells, significantly impairing their cytotoxic capacity (47). Thus, sCD160 serves as a soluble checkpoint, extending immune suppression beyond the immediate cell–cell interface. Notably, the release of sCD160 from melanoma cells seems constitutive and occurs through a classical protein secretion pathway, instead of proteolytic cleavage in NK cells. In contrast, a conference abstract reported that soluble CD160 (used as a blockade strategy of the CD160–HVEM interaction) augmented cytokine secretion and cytolytic activity by tumour-specific CD8^+^ T cells *in vitro*, highlighting that the functional meaning of “soluble CD160” depends on molecular format and experimental context; we grade that abstract as hypothesis-generating (50).

In stark contrast, a transmembrane isoform (CD160-TM) is generated via alternative splicing and is predominantly induced upon activation in NK cells (12). Unlike its GPI counterpart, CD160-TM possesses a distinct intracellular domain that recruits the Src-family kinase p56lck, initiating an ERK1/2-dependent signalling cascade that amplifies cytotoxic function. This expression pattern is tightly regulated; however, pathological deviations exist. For instance, in paroxysmal nocturnal haemoglobinuria (PNH), the loss of GPI-anchored proteins is associated with constitutive CD160-TM expression on circulating NK cells and spontaneous IFN-γ production (51). More recently, CD160-TM was identified as a neo-antigen on triple-negative breast cancer (TNBC) cells, suggesting that tumours may co-opt this activating receptor for intrinsic survival signalling (52). The role of the BTLA/HVEM/CD160 axis—and of soluble checkpoint molecules—in TNBC has recently been reviewed (53). This “Dr Jekyll and Mr Hyde” duality—where the same gene encodes a co-receptor, a soluble inhibitor, and a transmembrane activator—positions CD160 as a versatile rheostat of immune function (**Table 2**).

**Table 2:** Molecular formats of CD160.

Format	Origin/generation	Binds	Signalling and function	Main context	Evidence	Caveat
CD160-GPI	Predominant constitutive isoform; GPI-anchored, no cytoplasmic tail	HVEM (CRD1); HLA-C; MHC-I/CD1d (murine)	Lipid-raft/lateral signalling (CD2, PI3K→Akt/ERK, Syk); co-receptor on CD28^−^ CTL (p56lck/CD3ζ, activating); HVEM ligation on CD4^+^ T inhibitory	NK, CD8^+^/CD28^−^ T, CD4^+^ T, mast cells	established (18, 21, 24, 48, 49)	Net effect context-dependent; ectodomain shared with TM
CD160-TM	Alternative splicing; induced on NK activation	HVEM (weaker than GPI); tumour-cell expression	Cytoplasmic tail → p56lck → ERK1/2 → amplifies NK cytotoxicity (activating); proposed tumour-intrinsic signalling	Activated NK; aberrant in PNH; TNBC cells	strong preclinical (NK); hypothesis-generating (TNBC) (12, 36, 51, 52)	Shares ectodomain with GPI → needs isoform-discriminating reagents
Soluble CD160 (sCD160), natural	(i) Metalloprotease shedding from IL-15–activated NK; (ii) constitutive secretion by melanoma; mast-cell secretion	HVEM	“Soluble checkpoint” — inhibitory: impairs NK/CD8 cytotoxicity; melanoma immune escape; no diagnostic value in bladder-cancer serum	Serum; TME	limited correlative human (24, 46, 47, 54)	Meaning depends on source/format/assay; one conference abstract reported the opposite (50); distinct from engineered fusion
CD160-Ig (engineered)	Recombinant CD160 ectodomain–Ig fusion	HVEM	Blocks membrane CD160–HVEM costimulation; prolongs allograft survival (CD28-independent); conversely impaired NK tumour control/IFN-γ in one model	Experimental therapeutic	strong preclinical (55, 56)	Engineered ≠ natural sCD160

Reciprocal agonist tools that engage CD160 (for example HVEM-Ig) are HVEM-based reagents, not CD160 formats (13, 18). Rapoport 2026 is a TNBC axis review, not a fourth molecular format.

### 2.3 Dual identity and dual-function drug-design risk

The apparently contradictory descriptions of CD160 as either inhibitory or activating can be reconciled by recognising that its output is determined by three intersecting variables: the molecular isoform engaged, the cell type and differentiation state in which it is expressed, and the identity of the ligand.

At the level of isoform, the GPI-anchored form lacks an intracellular domain and signals through lateral membrane partners, acting as a co-receptor that augments TCR signalling on CD28-negative cytotoxic effector T cells (21) yet delivering inhibitory signals when it ligates HVEM on CD4^+^ T cells (18); the transmembrane form (CD160-TM), through its cytoplasmic tail and the p56lck–ERK1/2 axis, is activating in NK cells (12).

At the level of cell type, the same HVEM ligation can produce opposite outcomes in different lineages. HVEM specifically costimulates CD160 on effector NK cells—enhancing IFN-γ and TNF-α production and cytolysis via ERK1/2 and Akt—whereas HVEM engagement of BTLA is inhibitory (17). CD160 is thus best understood not as an intrinsic brake or accelerator but as a receptor whose valence is set by the cellular context.

At the level of differentiation state, CD160 marks distinct functional programmes along the exhaustion trajectory. The same CD160^+^PD-1^+^ surface phenotype can identify advanced dysfunction in some chronic-antigen settings (57) yet mark polyfunctional, cytolytic, self-renewing CD8^+^ T cells in others, including protective programmes described in chronic viral infection (58). No oncology, autoimmunity or transplantation dataset currently contrasts these two valences within a single human cohort; we therefore retain this compressed observation as a mechanistic constraint, not as an infection-immunology narrative. In CRC, CD160 signalling is required to sustain the stem-like progenitor-exhausted pool whose loss precipitates terminal exhaustion (14).

We therefore propose that CD160 be interpreted through an “isoform × compartment × ligand” framework rather than a single directional label. Where current data are insufficient to assign a definitive mechanism—particularly the relationship between progenitor-exhaustion programmes and terminal dysfunction—we regard the competing models as unresolved rather than settled. Because valence is set by isoform and lineage, a systemic antibody to the shared ectodomain cannot be assumed to act in one direction—a dual-function drug-design constraint we return to in the Discussion (Section 6).

## 3 Immuno-oncology

If cancer is a disease of pathological tolerance, the translational goal in this chapter is to break that tolerance without collapsing the progenitor-exhausted pool that seeds durable responses, and without silencing NK-mediated tumour control. Autoimmunity and transplantation invert this logic (Sections 4 and 5) (**Figure 2**).

**Figure 2:**
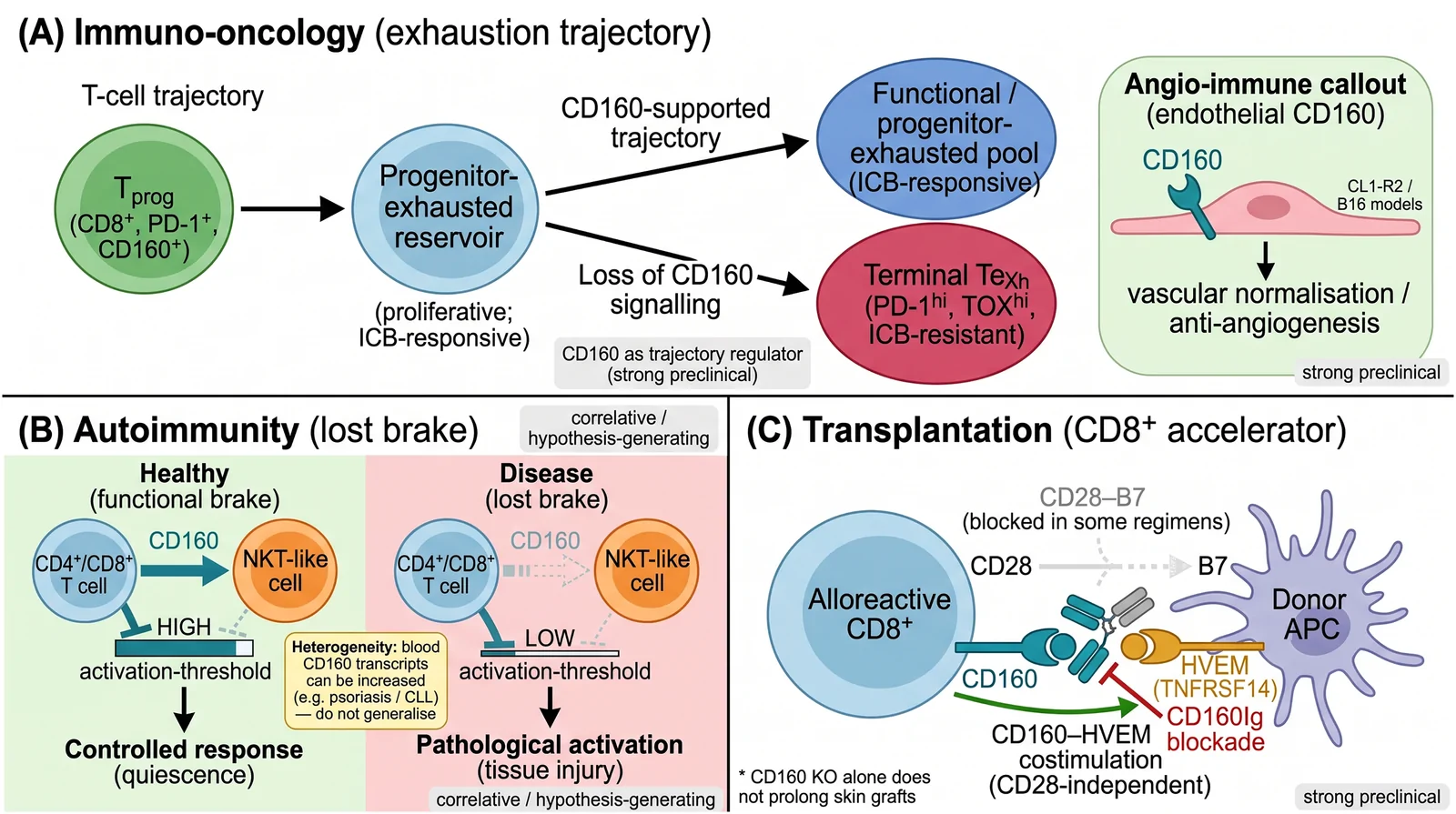
Reciprocal paradigm across three disease settings. Original schematic (redrawn from the authors’ AI artwork; not adapted from published diagrams). Three disease panels are drawn: **(A)** immuno-oncology, **(B)** autoimmunity, **(C)** transplantation. Vascular biology is the green angio-immune callout inside **(A)**, not a fourth disease panel. **(A)** Immuno-oncology (exhaustion trajectory, with angio-immune callout): A progenitor CD8^+^ T cell (PD-1^+^, CD160^+^) feeds a progenitor-exhausted reservoir. A CD160-supported trajectory maintains a functional/progenitor-exhausted, ICB-responsive pool; loss of CD160 signalling is linked to terminal Texh (PD-1hi, TOXhi, ICB-resistant). The green callout shows endothelial CD160 (CL1-R2/B16 models) leading to vascular normalisation/anti-angiogenesis. Graded strong preclinical, not speculative. **(B)** Autoimmunity (lost brake): Healthy (functional brake) versus disease (lost brake) cartoons with CD4^+^/CD8^+^ T cells and NKT-like cells. High CD160 raises the activation threshold (controlled response/quiescence); low CD160 lowers it (pathological activation/tissue injury). A heterogeneity box notes that blood CD160 transcripts can be increased (e.g. psoriasis/CLL) — do not generalise. Graded correlative/hypothesis-generating. **(C)** Transplantation (CD8^+^ accelerator): Alloreactive CD8^+^ T cell versus donor APC. CD28–B7 is marked blocked in some regimens; CD160–HVEM costimulation (CD28-independent) is intercepted by CD160Ig blockade. CD160 knockout alone does not prolong skin grafts. Evidence levels: CD160 as a regulator of the exhaustion trajectory **(A)** is strong preclinical; the lost-brake model **(B)** is hypothesis-generating (correlative/genetic data) with an explicit heterogeneity caveat; transplantation blockade **(C)** is strong preclinical; the angio-immune quiescence coupling (callout in A) is strong preclinical.

### 3.1 Key genetic and genomic links

Germline oncology evidence for CD160/HVEM is limited correlative human, not established causality. In clear-cell renal-cell carcinoma (ccRCC)—a disease of direct relevance to a urology department—heterozygosity at *CD160* rs2231375 increased risk (odds ratio 1.47 in the reported comparison), and co-carriage with *HVEM* rs2234167 AG approximately doubled risk; *HVEM* rs1886730 associated with overall survival (59). These associations require independent replication. A CD160 promoter AML-1 (RUNX1) site has been described and is a plausible transcriptional control point, but it is not a cancer-risk variant in its own right (60). Plasma extracellular-vesicle (EV) *CD160* transcript abundance predicted immunochemotherapy outcome in lung adenocarcinoma, which we grade as limited correlative human/emerging biomarker rather than a genetic link (61). Blood *CD160* methylation has been reported in a breast-cancer case–control series; we treat that finding as hypothesis-generating and do not quote an odds ratio from that Chinese case–control series (n=272) (62). We do not cite retracted lncRNA-CD160 literature. Overall, oncology genetics of this axis is thin: SNPs and liquid-biopsy transcripts annotate risk or treatment association, they do not yet define a therapeutic genotype.

### 3.2 Which cells are most relevant

In tumours, CD160 annotates more than one programme, and the programmes must not be collapsed into a single “checkpoint-positive” label (cross-reference **Table 1**).

On CD8^+^ tumour-infiltrating lymphocytes (TILs), CD160 can mark a constrained activation trajectory. In pancreatic cancer, tumour-infiltrating CD8^+^ T cells were enriched for a CD160^+^ subset that expressed higher PD-1/TIM-3 and showed higher *ex vivo* cytokine readouts yet displayed reduced proliferative capacity and cytotoxicity upon stimulation and correlated with poorer survival (63). In gastric cancer, CXCL13^+^CD160^+^CD8^+^ T cells promote tertiary lymphoid structure (TLS) formation and associate with immunotherapy benefit—an anatomy-level, not purely exhaustion-level, reading of the same marker (64). These observations are not necessarily contradictory; they underscore that CD160 should be interpreted within the differentiation state and co-inhibitory network that collectively shape exhaustion.

Zheng et al. demonstrated that in CRC, CD160 signalling is essential for maintaining the stem-like, progenitor-exhausted CD8^+^ T cell pool (often characterised by Tcf1 expression) (14). Contrary to a blockade-centric reading, it is the loss of CD160 signalling that precipitates terminal exhaustion and resistance to PD-1 blockade. These CD160^+^ progenitor cells retain the capacity for self-renewal and proliferation, serving as the resource that replenishes the effector pool upon checkpoint blockade. Consequently, the absence of CD160 dictates a state of deep, irreversible exhaustion that persists despite anti-PD-1 therapy. This is a single-study, strong-preclinical claim: CD160 acts not as a brake to be released, but as a signal that helps preserve the progenitor pool and may need to be maintained. We do not claim that CD160 blockade synergises with PD-1. The resistance observed in CD160-low tumours is likely rooted in the epigenetic landscape, where the loss of CD160-mediated signalling allows the transcription factor thymocyte selection-associated high-mobility-group box protein (TOX) to enforce an irreversible epigenetic scar, locking cells into a non-responsive state (65).

In stark contrast to its dual role on T cells, CD160 can act as an activating receptor in the NK compartment, with isoform- and context-dependent outputs. In human NK cells, engagement of CD160 by HLA-C—constitutively expressed on K562 cells—was shown to trigger cytotoxicity through direct CD160–HLA-C interaction; inhibitory CD158b (killer-cell immunoglobulin-like receptor, KIR) receptors partially interfered with this CD160-dependent lysis pathway (15). Complementing this, blockade of CD160 using CL1-R2 markedly reduced NK-92-mediated cytolysis against leukaemic targets, and CD160 expression on peripheral blood NK cells was reduced in patients with acute myeloid leukaemia (AML)/CLL and in allogeneic haematopoietic stem cell transplantation (allo-HSCT) recipients (66). Mechanistically, CD160-TM is selectively induced upon NK activation and can recruit p56lck to trigger ERK1/2-dependent signalling, consistent with an amplification role in degranulation (12). Reduced NK CD160 in hepatocellular carcinoma (HCC) associates with impaired NK function and poorer clinical outcome (67). Together, these observations support CD160 as a functional axis that can couple or uncouple NK cytotoxicity and cytokine outputs depending on ligand context and differentiation state.

The suppressive arm is not confined to lymphocytes. Mast cells constitutively express and secrete CD160-GPI, providing a local source of soluble CD160 capable of engaging HVEM on CD8^+^ T cells and dampening cytotoxic activity (24). HVEM-high circulating monocytes in PDAC are a complementary, ligand-side correlate of poor outcome (32). Most surprisingly, CD160-TM is expressed directly on TNBC cells, suggesting that tumours may repurpose this molecule for tumour-intrinsic signalling or cell–cell communication (52, 53). Malignant B cells in CLL/HCL express CD160 as a tumour-specific antigen distinct from T-cell CD160 (26, 27). Lymphocytes, mast cells, endothelium (Section 3.5), monocytes and tumour cells may therefore exploit distinct CD160 or HVEM configurations to maintain a sanctuary of immune regulation.

### 3.3 *In vivo* models

In tumour-bearing mice, CD160 upregulation on CD8^+^ T cells tracked with reduced proliferation, perforin and IFN-γ, and blocking the CD160–HVEM interaction partially restored these functions (68). Age-associated transcriptional constraints intersect this axis: blocking BTLA/CD160 signalling during vaccination partially reverted age-related gene-expression defects in antigen-specific CD8^+^ T cells, although in human ageing CD244 (2B4) rather than CD160 appeared to be the dominant functional driver of senescence-like dysfunction (69, 70). We compress this to an honesty signal: mouse vaccination data do not automatically transfer to human immunosenescence.

Zheng and colleagues used *Cd160* knockout, MSI-high and inflammation-induced CRC models, and adoptive transfer of CD160^+^CD8^+^ T cells. Transfer of CD160^+^CD8^+^ T cells inhibited tumour growth; knockout accelerated it; in MSI-high and anti-PD-1-resistant settings, CD160^+^CD8^+^ T cells improved anti-PD-1 efficacy by increasing tumour-infiltrating progenitor-exhausted T cells (14). We state these as the model types used in that study. We do not aggregate them as “four independent mouse CRC models,” and we do not invent effect sizes beyond the paper’s qualitative claims. A News & Views commentary is not an independent experimental replicate (71).

In a humanised HVEM/BTLA double knock-in, an antagonistic anti-HVEM antibody (HVEM18-10) that blocks HVEM engagement by BTLA and CD160 reduced HVEM-positive tumour growth, decreased exhausted CD8^+^ T cells and regulatory T cells, increased effector-memory CD4^+^ T cells, synergised with PD-L1 blockade, and left a minority of rejector mice protected against rechallenge (approximately 20% in the published abstract) (38). This is the strongest *in vivo* hook for hub targeting, but it is a mouse model expressing human molecules, not a clinical trial.

An honest negative is mandatory for dual-function risk. In CD160-deficient mice, control of NK-sensitive tumours was compromised because of markedly reduced NK-derived IFN-γ, despite preserved cytotoxicity; soluble CD160-Ig similarly impaired tumour control and IFN-γ production (55). Any agent that occupies CD160 or its HVEM interface may therefore subtract innate control even if it relieves T-cell inhibition. CL1-R2 plus cyclophosphamide in B16 melanoma is discussed as vascular normalisation in Section 3.5 (72). The anti-CD160-TM antibody 22B12 has *in vitro* activity against TNBC spheroids; *in vivo* tumour-growth effect sizes were not uniformly reported and are not quoted here (52).

### 3.4 Translational and clinical trial data

State of play. No direct anti-CD160 antibody has a verified oncology investigational new drug application or interventional trial in the bibliography of this manuscript. While direct anti-CD160 candidates remain in advanced preclinical development, other components of the HVEM network are actively undergoing human clinical testing. Developers have prioritised the hub: tifcemalimab (icatolimab; TAB004; JS004) is an anti-BTLA mAb and does not bind CD160 (73–76). Antagonistic anti-HVEM antibodies block both BTLA and CD160 engagement of the shared CRD1 surface. Demerlé anti-HVEM18–10 is an academic reagent tested in humanised HVEM/BTLA mice (38). Fully human 4CB1 (4C Biomed; Bareli, Barshack, Schachter, Markel and colleagues, Sheba Medical Center) is a distinct anti-HVEM mAb reported to block HVEM–BTLA and HVEM–CD160; composition-of-matter is described in patent WO2020222235A1 (77, 78). We do not assign 4CB1 a numbered clinical-trial identifier or Phase I status: the 2026 Molecular Cancer Therapeutics report is preclinical and ex-vivo, and no NCT was found. Preclinical anti-CD160 assets that justify the phrase “advanced preclinical” include CL1-R2, humanised ELB01101, 22B12, CD160-Ig, and CD160-derived peptide A5, which blocks BTLA/HVEM and HVEM/LIGHT *in vitro* (79) (**Table 3**).

**Table 3:** Clinical and preclinical agents in the HVEM network.

Agent (INN/code)	Target/mechanism	Modality	Development stage	Trial ID	Indication/setting	Line of therapy	Efficacy (only if sourced)	Safety (only if sourced)	Refs
Tifcemalimab (icatolimab; TAB004; JS004)	Anti-BTLA mAb; does not bind CD160	Human mAb (Junshi/Coherus)	Phase I lymphoma; Phase I/II previously treated lung cancer; Phase 3 LS-SCLC consolidation (ongoing)	NCT04477772 (lymphoma, Song 2025). NCT05000684 (Phase I/II previously treated lung, Cheng 2025). NCT06095583 (Phase 3 LS-SCLC consolidation; not Cheng 2025)	Relapsed/refractory lymphoma; previously treated NSCLC and SCLC; LS-SCLC consolidation after CRT (Phase 3)	Later-line/previously treated; not labelled first-line	Lymphoma: n=25 monotherapy + 46 combination; no DLT; MTD not reached; RP2D 200 mg. Lung: NSCLC ORR 4.3% (n=24); SCLC ORR 35.0% (n=43); 200 mg + toripalimab 240 mg Q3W	Lymphoma grade 3/4 TRAE 12.0% (monotherapy)/32.6% (combination); no fatal AE	(75, 76) (73)
Anti-4CB1	Fully human anti-HVEM; blocks HVEM–BTLA and HVEM–CD160	mAb, 4C Biomed; Galore-Haskel et al. (Bareli co-author), Sheba Medical Center	Preclinical + ex-vivo (49 tumours); not Phase I	None found	Solid tumours (preclinical/ex-vivo)	n/a	Ex-vivo cytotoxicity increased in 28.5% of 49 samples; *in-vivo* activity ± anti-PD-1	Not a clinical safety dataset	(77)
Patent WO2020222235A1	Composition-of-matter for anti-HVEM (4CB1 lineage)	Patent	n/a	n/a	n/a	n/a	n/a	n/a	(78)
Anti-HVEM18-10 (Demerlé)	Antagonistic anti-HVEM; blocks BTLA and CD160 engagement	Mouse/humanised-model mAb	Preclinical (humanised HVEM/BTLA KI mice)	None	HVEM^+^ tumours; ± anti-PD-L1	n/a	Reduced tumour growth; PD-L1 synergy; ~20% rechallenge protection (abstract)	Not a clinical safety dataset	(38)
CL1-R2	Anti-CD160; endothelial new vessels; also blocks NK lysis	mAb	Preclinical (ocular; B16 + cyclophosphamide)	None	Vascular/melanoma models	n/a	B16 vascular normalisation with cyclophosphamide	Not clinical	(72)
ELB01101	Humanised anti-CD160	mAb	Advanced preclinical (ophthalmology)	None	Ocular neovascularisation (rabbit/monkey)	n/a	~50% reduction in clinically relevant ChNV lesions in monkeys	Not oncology	(82)
22B12	Anti-CD160-TM	mAb	Preclinical	None	CD160-TM^+^ TNBC	n/a	*In vitro* spheroid signal loss; *in vivo*	Not clinical	(52)
CD160Ig	Engineered CD160 ectodomain–Ig; blocks membrane CD160–HVEM costimulation	Fusion protein	Preclinical	None	Cardiac/skin allograft (murine)	n/a	CD28−/− MST 30 vs 12 days; CD4−/− MST 56 vs 34 days	Impaired NK tumour control in Tu 2015	(56)
HVEM-Ig	Agonist/ligand tool for CD160 restoration	Fusion protein	Reagent/hypothesis-generating	None	Autoimmunity (conceptual)	n/a	None clinical	Immunosuppression risk	(13) (18)
Peptide A5	CD160-derived peptide; bidirectional BTLA/HVEM and HVEM/LIGHT blockade	Peptide	*In vitro*	None	Checkpoint-blockade concept	n/a	*In vitro* only	Unknown	(79)
Sirolimus (context only)	mTOR inhibitor; not HVEM-directed	Approved	Approved	n/a	Renal transplant	Maintenance	Bai 2013: raises BTLA/CD160, lowers LIGHT, raises HVEM on CD4^+^ Tregs	Known	(93)
Belatacept (context only)	CTLA4-Ig; CD28 costimulation blockade	Approved fusion	Approved	n/a	Kidney transplant	Maintenance	Standard of care	Known	(91)

Development stages are those supported by the cited sources at the time of writing. Direct anti-CD160 candidates have not entered verified human oncology, autoimmunity or transplantation trials. Tifcemalimab is an anti-BTLA antibody, not an anti-CD160 antibody. Anti-4CB1 and Demerlé anti-HVEM18–10 are related in mechanism (HVEM hub blockade) but are distinct agents; they must not be conflated. Clinical cells are those supported by the cited primary papers; 4CB1 has no NCT.

Belatacept is not an HVEM-network agent; it is mentioned only as the CD28-blockade resistance context.

Efficacy. For tifcemalimab, first-in-human Phase I lymphoma data (NCT04477772; n=25 monotherapy plus n=46 with toripalimab) reported no dose-limiting toxicity, an unreached maximum tolerated dose, and a recommended Phase 2 dose of 200 mg, and a Phase I/II lung combination study (NCT05000684) reported ORR 4.3% in NSCLC (n=24) and 35.0% in SCLC (n=43) at tifcemalimab 200 mg plus toripalimab 240 mg every 3 weeks (75, 76). NCT06095583 is a separate Phase 3 limited-stage SCLC consolidation study of toripalimab alone or with tifcemalimab (JS004/TAB004) after chemoradiotherapy (sponsor Shanghai Junshi Bioscience) and is not the Cheng 2025 Phase I/II dataset. For anti-HVEM, the strongest published efficacy is preclinical: reduced HVEM^+^ tumour growth and PD-L1 synergy in the Demerlé model (38). Direct anti-CD160 has no clinical efficacy dataset. A key translational readout is not simply “more IFN-γ,” but maintenance or expansion of the progenitor-exhausted compartment that seeds response to checkpoint therapy (7, 14). In melanoma, tumour-associated sCD160 has been linked to impaired NK-cell function and immune escape (47); the more testable strategy is sCD160-aware intervention in patients with demonstrably elevated sCD160, rather than blanket anti-CD160 receptor blockade.

Safety. No clinical NK-toxicity signal has been reported for anti-CD160 antibodies because no such antibody has reached the clinic. The pathway risk is nonetheless explicit: CD160-Ig itself impaired NK-mediated tumour control in mice (55), and HVEM costimulates NK CD160 (17). Hub blockade may precipitate immune-related adverse events (irAEs) analogous to established ICB, and will also perturb the LIGHT costimulatory arm (3, 37). In the lymphoma Phase I study, grade 3/4 treatment-related adverse events occurred in 12.0% of monotherapy and 32.6% of combination recipients; no fatal adverse events were observed (75, 76).

Population and line of therapy. Hypothesis-generating enrichment markers include CD160-low/terminally exhausted tumours (14), elevated serum sCD160 (47), CD160-TM^+^ TNBC (52), CD160^+^ CLL/HCL (26), plasma EV-*CD160* (61), and CXCL13^+^CD160^+^ TLS density (64). Line of therapy for tifcemalimab in the cited primaries is relapsed/refractory lymphoma and previously treated advanced lung cancer; we do not relabel it as first-line. Direct anti-CD160 has no definable line of therapy. In TNBC, CD160-TM expression on tumour cells enables Fc-enabled depletion or antibody–drug conjugate (ADC)-style concepts only if isoform specificity is engineered and confirmed (52).

### 3.5 The vascular arm: endothelial CD160 and angio-immune quiescence

This strand is cross-cutting—it is relevant to inflammatory angiogenesis in autoimmunity and, as a hedged possibility, to chronic allograft vasculopathy—but its strongest *in vivo* data are tumour data, so we grade and discuss it here rather than as a fourth disease chapter. Evidence grade for vascular/endothelial biology: strong preclinical.

CD160 expression on endothelial cells links immune regulation to tissue-level physiology: soluble HLA-G1 can bind endothelial CD160 and trigger apoptosis, thereby inhibiting angiogenesis (25). Conceptually, these observations support an angio-immune quiescence programme in which a single receptor axis can simultaneously restrain effector T-cell function and limit vascular expansion (72, 80). This coupling is mechanistically attractive for inflammatory settings where pathological angiogenesis and immune activation reinforce each other (for example rheumatoid-arthritis pannus) (81), but in tumours it may be co-opted to stabilise an immunosuppressive niche.

The strongest proof-of-principle for this vascular arm comes from *in vivo* targeting studies: an anti-CD160 mAb (CL1-R2) preferentially recognised endothelial CD160 on newly formed (tumour) vessels, inhibited pathological neovascularisation in ocular models, and—when combined with cyclophosphamide—induced regression and normalisation of B16 melanoma vasculature consistent with improved vessel function and drug access (72). A humanised anti-CD160 (ELB01101) was a potent inhibitor of ocular neovascularisation in rabbit and monkey models, including when combined with bevacizumab; in the monkey choroidal-neovascularisation model, ELB01101 reduced clinically relevant lesions by approximately 50% versus vehicle (82). CD160 expression in human retinal vessels associates with retinal neovascular diseases (83). A 2024 meeting abstract reported that anti-CD160 reduced fibroblast growth factor-2 (FGF-2)-induced tube formation in activated human umbilical-vein endothelial cells (HUVECs) without inducing apoptosis, supporting a normalisation rather than a purely cytotoxic endothelial mechanism; we grade the abstract as strong preclinical/pending full paper (84).

We close with two translational cautions. First, targeting endothelial CD160 to normalise tumour vasculature could, in principle, also affect physiological angiogenesis and wound healing (25, 72). Second, a dedicated endothelial-CD160 paper in chronic allograft vasculopathy was not found; relevance to transplantation is therefore hypothesis-generating unless a primary source is identified. The same biology is already cited for rheumatoid pannus in autoimmunity, so the vascular arm is not oncology-exclusive—it is oncology-led.

## 4 Autoimmunity

If immuno-oncology is pathological tolerance to be broken, autoimmunity is physiological tolerance lost. The translational goal is restoration of an inhibitory pairing, not its blockade—except in those diseases and compartments where CD160 is upregulated, where a systemic agonist could be the wrong tool.

### 4.1 Key genetic links

The link between CD160 insufficiency and autoimmunity is not merely transcriptomic but genetic. He et al. identified CD160 as the most significantly downregulated co-signalling gene across multiple autoimmune conditions, including systemic lupus erythematosus (SLE) and Graves’ disease (85). A subsequent case–control study found that *CD160* rs744877 is associated with Graves’ disease, with a protective direction of effect (allele odds ratio = 0.84; dominant odds ratio = 0.74). There was no association with Hashimoto’s thyroiditis, and none for rs3766526 (86). Genetic variants in this axis may therefore shift the threshold for thyroid autoimmunity in a disease-specific way. Evidence grade: limited correlative human.

### 4.2 Which cells are most relevant — and why the direction of change is not uniform

If cancer is a disease of too much tolerance often mediated by the overexpression of checkpoints, autoimmune diseases represent the reciprocal failure of these restraints. That “lost brake” framing is a starting hypothesis, not a uniform rule.

Importantly, autoimmunity can also be accompanied by a broader, context-dependent remodelling of immunoregulatory gene programmes rather than a uniform loss of inhibitory signals. In a peripheral-blood quantitative reverse-transcription polymerase chain reaction (qRT-PCR) study comparing CLL, psoriasis and healthy volunteers, BTLA, CD160 and sialophorin (SPN; CD43, gene *SPN*) transcripts were reported as overexpressed in both CLL and psoriasis relative to controls, with additional co-variation between CD160 and TIM-3 expression (87).

Skin CD4^+^CD160^+^ T cells infiltrate inflammatory dermatoses and a CD4^+^CD160^+^CD158k^+^ subset is detectable in normal and mycosis-fungoides skin (19, 20). IEL CD160–epithelial HVEM signalling, although characterised in injury-repair models, is a plausible mucosal pairing in intestinal autoimmunity (22). NKT cells are a second innate-like compartment in which CD160 is a negative regulator (Section 4.3).

It is important to note that the “lost brake” model does not apply uniformly across autoimmune diseases, and the direction of CD160 dysregulation is itself context-dependent. Whereas CD160 is downregulated in several systemic conditions (85), Skórka et al. reported increased BTLA, CD160 and SPN transcripts in both CLL and psoriasis relative to controls (87). This apparent discrepancy can be attributed to several factors that should be considered when interpreting expression data: the compartment sampled (lesional tissue versus circulating blood), the activation and differentiation state of the cells analysed, the cellular composition of bulk samples (which average across shifting subset proportions), the stage of inflammation, and the effects of ongoing therapy. Consistent with a compartment- and state-dependent interpretation, a recent serum study in bladder cancer found that soluble CD160 clustered with soluble HVEM and showed no independent diagnostic value, a pattern attributed to shedding during T-cell exhaustion rather than to a fixed directional change (54). We therefore frame CD160 dysregulation in autoimmunity as a context-dependent remodelling of inhibitory cosignalling rather than a uniform loss, and caution against generalising from any single disease or compartment.

### 4.3 *In vivo* models

The protective role of CD160 extends beyond the adaptive immune system to innate-like lymphocytes. Kim et al. identified CD160 as an essential negative regulator of NKT cells. CD160 is not required for NKT development; in a model of acute hepatic injury, however, CD160-deficient mice exhibited exacerbated liver damage driven by hyperactivated NKT cells with enhanced IFN-γ, TNF and IL-4 production, and anti-BTLA further aggravated injury in the knockout, indicating non-overlapping function with BTLA (88). This indicates that CD160 functions as a firewall against cytokine storms mediated by innate lymphocytes. HVEM muteins that preserve immunoglobulin-ligand (BTLA/CD160) engagement while disabling LIGHT ameliorate liver inflammation *in vivo*, showing that the inhibitory arm can be isolated genetically (37). Adoptive transfer of CD160-sufficient IELs rescues chemotherapy-induced intestinal injury in *Rag1*-deficient mice, a regeneration model rather than a classical autoimmune model, but it is the cleanest *in vivo* demonstration that IEL CD160–epithelial HVEM is necessary for barrier recovery (22). What these models do **not** show is that a systemic CD160 agonist treats human autoimmunity.

### 4.4 Translational and clinical trial data

Consequently, the therapeutic imperative in autoimmunity is, in selected contexts, to restore this brake. As reviewed by Piotrowska et al., CD160 represents a candidate for restoration therapy (13). By using CD160 agonists (for example HVEM-Ig) to re-engage this inhibitory pathway on hyperactivated CD4^+^ T cells and NKT cells, such agents could, in principle, promote a more quiescent state in these autoreactive clones, dampening inflammation without broad immunosuppression (18). We keep the hedge: this is a principle, not a clinical result.

Efficacy. No CD160-directed agent is in clinical testing for autoimmunity in this repository. There is therefore no efficacy dataset.

Safety. Restoring an inhibitory brake carries the generic risks of immunosuppression, including impaired antitumour and antiviral surveillance (3). Heterogeneity means a systemic agonist could be actively wrong in psoriasis-like high-CD160 blood-transcript states (87).

Patient population. If a restoration strategy is attempted, enrichment should favour CD160-low compartments, and tissue rather than blood alone (Section 4.2). Psoriasis and CLL blood overexpression are explicit counter-examples.

Line of therapy. Cannot be defined.

The nearest translational path is not anti-CD160 at all, but agonist anti-BTLA epitope work already at structural resolution (45), or HVEM-Ig as a reagent-stage agonist (13). Endpoints should include tissue-level readouts (vascular remodelling, barrier repair), not immune phenotypes alone (23, 25). This chapter therefore closes by pointing back to oncology (where hub blockade is being tested) and forward to transplantation (where the same ectodomain is a CD8^+^ accelerator to be blocked, not restored).

## 5 Transplantation

The third face of the reciprocal paradigm is transplantation: here CD160 functions as a CD28-independent accelerator of alloreactive CD8^+^ T cells. The goal is blockade, not restoration.

### 5.1 Key genetic and genomic links

Beyond T-cell costimulation, CD160 appears in effector programmes implicated in antibody-mediated rejection (ABMR). An *in vitro*-derived antibody-dependent cellular cytotoxicity (ADCC) gene set (including CD160) was overexpressed in renal biopsies from patients with ABMR, including C4d-negative cases, supporting a complement-independent, cytotoxic injury axis (89). This is a **transcriptomic** association in graft tissue, not a germline finding. A candidate-gene association study using next-generation sequencing (NGS) across HVEM/LIGHT/BTLA/CD160 variants in 200 renal transplant recipients did **not** identify single-nucleotide polymorphism (SNP) associations with ABMR risk, suggesting that (at least in that cohort) pathway genetics may be less informative than dynamic immune states (90). We keep this negative result prominent: it is the genetic-link beat, honestly empty of a risk allele.

### 5.2 Which cells are most relevant

In organ transplantation, the role of CD160 shifts from a brake to a critical accelerator of rejection, particularly within the CD8^+^ compartment. Unlike its inhibitory role on CD4^+^ T cells, CD160 functions as a costimulatory receptor on alloreactive CD8^+^ cytotoxic T cells, driving their expansion and effector function (91). This pathway becomes especially dominant when the classical CD28 costimulatory signal is blocked, serving as a candidate resistance mechanism to belatacept (CTLA4-Ig) therapy. The same study, however, found that CD160 deficiency alone did not significantly prolong skin allograft survival, and that CD160-knockout NK cells remained proficient at rejecting allografts (91). The belatacept-resistance hypothesis therefore remains translationally attractive but is hedged by that negative survival result. Alloreactive CD8^+^ T cells, not NK cells, are the relevant CD160^+^ effectors for graft prolongation strategies; IELs and endothelium are relevant for tissue endpoints (Sections 1.3 and 3.5) rather than as the primary rejection engine.

### 5.3 *In vivo* models

Antibody-mediated targeting of CD160 can modulate allogeneic cytotoxicity *in vivo*: in an MHC class I-mismatched skin-graft model, anti-CD160 combined with anti-CD40L to limit CD4^+^ T cell help prolonged graft survival and attenuated effector CD8^+^ T cell responses (92). D’Addio et al. provided the proof-of-concept for a ligand-trap approach. They developed a soluble CD160Ig fusion protein that binds to HVEM, effectively blocking the interaction with membrane-bound CD160 on T cells. In a fully mismatched cardiac transplant model, CD160Ig treatment significantly inhibited alloreactive CD8^+^ T cell proliferation and prolonged allograft survival, with effects being most pronounced in CD28-deficient recipients (56). Median survival was 30 versus 12 days in CD28-deficient recipients (p=0.002) and 56 versus 34 days in CD4-deficient recipients (p=0.01), taken from Zotero PDF notes on the PLoS ONE primary. This establishes CD160 as a CD28-independent costimulatory pathway. Del Rio 2022 used CRISPR/Cas9 exon-2 deletion and F1 transfer to show reduced alloreactive CD8^+^ expansion without a significant single-agent survival benefit, which is the result that must travel with the D’Addio success (91). What the models do not show is that CD160Ig is ready for a transplant trial.

### 5.4 Translational and clinical trial data

Therefore, unlike autoimmunity where the goal is restoration, the strategy in transplantation is blockade: using CD160Ig or antagonists to dismantle the costimulatory drive of alloreactive CD8^+^ T cells. CD160Ig-type approaches that engage HVEM can be framed as tolerance engineering—particularly attractive when rejection pathways bypass CD28 (56, 91).

Efficacy. No CD160- or HVEM-directed agent has been tested in a transplantation trial in this repository. There is no clinical efficacy dataset.

Safety. CD160Ig impaired NK tumour control in an oncology model (55); whether that trade-off is acceptable after solid-organ transplantation is unknown. Endothelial targeting could theoretically affect wound healing and, hypothetically, vasculopathy (Section 3.5).

Patient population. The most coherent enrichment hypothesis is recipients in whom CD28 costimulation is already blocked (belatacept/CTLA4-Ig) or genetically less dominant (the CD28-deficient murine analogue) (91).

Line of therapy. Frame as induction versus maintenance versus ABMR rescue: none is established.

The only human pharmacodynamic datum in this chapter is not an HVEM-directed drug. In allo-renal recipients receiving a sirolimus-based regimen, BTLA and CD160 expression on circulating T cells increased while LIGHT decreased, alongside higher HVEM on CD4^+^ regulatory T cells, consistent with a systemic shift toward inhibitory cosignalling that could support tolerance maintenance (93). Sirolimus is an approved mechanistic target of rapamycin (mTOR) inhibitor; we cite it as context that an already-used maintenance drug tilts the switchboard, not as an HVEM-network agent. Combination with costimulatory blockade remains the D’Addio/Del Rio hypothesis (strong preclinical). This chapter therefore points back to immuno-oncology, where hub antibodies are closer to the clinic, and to autoimmunity, where the same fusion proteins would be used with the opposite intent.

## 6 Discussion

The therapeutic opportunity around CD160 is real, but the decision point is not simply “block versus agonise.” The actionable unit is the CD160 form (GPI/TM/soluble) in a defined compartment (T cell/NK/endothelium/tumour/IEL/malignant B cell)—because each combination implies a different mechanism, efficacy ceiling and safety risk. Across immuno-oncology, autoimmunity and transplantation, the common failure mode is conceptual rather than technical: CD160 is treated as a single checkpoint target, when it behaves as a platform whose biological meaning is defined by form, compartment and disease goal.

Reciprocal synthesis. The pathological exhaustion that shields tumours is the physiological tolerance lost in autoimmunity and the CD28-independent acceleration of rejection in transplantation. In CRC, CD160 helps preserve a Tcf1^+^ progenitor-exhausted CD8^+^ pool; losing that signal deepens PD-1-refractory exhaustion (14). In Graves’ disease and several systemic autoimmunities, CD160 is among the most downregulated co-signalling genes, yet psoriasis and CLL blood transcripts move in the opposite direction (85, 87). In the allograft, CD160 costimulates alloreactive CD8^+^ T cells when CD28 is weak, and CD160Ig prolongs cardiac grafts most clearly in that setting—yet knockout alone does not significantly prolong skin grafts (56, 91). The reciprocal paradigm therefore survives the three-part structure: the same molecular codes are read with opposite therapeutic signs. The path to restoring tolerance may still be paved by the mechanisms of tumour survival, provided those mechanisms are not over-generalised.

Network redundancy. Because HVEM binds BTLA, CD160 and LIGHT, drug developers have prioritised targeting BTLA (via tifcemalimab) or HVEM directly (anti-HVEM18-10; 4CB1), as blocking a single hub molecule can disrupt multiple inhibitory pathways at once (38, 73, 77, 80). While direct anti-CD160 candidates remain in advanced preclinical development, other components of the HVEM network are actively undergoing human clinical testing. The counter-argument is equally structural: hub blockade also perturbs the LIGHT costimulatory arm. HVEM muteins show that the LIGHT and immunoglobulin-ligand arms are separable *in vivo* (37). Hub blockade is therefore not equivalent to cleanly blocking three inhibitory pathways; it is a bet that the net of removing two immunoglobulin ligands outweighs the cost of disturbing LIGHT. Because HVEM is a shared hub, any agent that modulates HVEM globally risks off-target effects across the entire network rather than the intended single interaction (44).

Dual-function risk. Because CD160 is inhibitory on T cells but activating on NK cells via CD160-TM, designing a systemic antibody requires precise engineering to avoid accidentally shutting down innate NK-mediated tumour clearing (12, 17, 18). This is not a theoretical worry: CD160-Ig, the same format that prolongs cardiac allografts, impaired NK tumour control and IFN-γ in mice (55, 56). CD160-GPI and CD160-TM share an identical ectodomain, so discriminating reagents are required to avoid engaging the unintended isoform, and “CD160 positivity” in tissue cannot be equated with an actionable target without isoform-resolved detection (36, 52). Engineering responses include isoform-discriminating mAbs (22B12), Fc designs that spare NK, hub targeting that does not agonise NK CD160, and local delivery (ocular CL1-R2/ELB01101) (52, 72, 82).

What not to do with a systemic anti-CD160. Do not treat CD160 positivity as an actionable target without isoform and lineage. Do not blockade CD160 in CRC-like settings where the molecule preserves progenitor-exhausted cells (14). Do not restore the brake systemically in diseases where CD160 is upregulated in the sampled compartment (psoriasis/CLL blood transcripts) (87). Do not ignore endothelial CD160 (wound healing and physiological angiogenesis) (25). Agonistic strategies intended to restore inhibitory tone in autoimmunity or transplantation carry immunosuppression risk, whereas blocking strategies intended to reinvigorate antitumour immunity may precipitate irAEs (3). Naturally shed sCD160 may act as a circulating decoy that alters the exposure and dosing of antibody-based therapeutics (46, 47). Each of these considerations reinforces the central argument that CD160-directed therapy must be specified by form, compartment and interaction geometry before clinical translation.

Isoform specification is not a detail to be cleaned up later; it is the starting point. A programme built to neutralise tumour-associated sCD160 should not be evaluated with the same assumptions as a programme aiming to modulate cell-bound CD160 signalling, and a tumour-targeting strategy that relies on CD160-TM must be judged by the rigour of its isoform discrimination rather than by CD160 positivity in the abstract. Compartment specification must be explicit because immune and vascular contexts imply different mechanisms and safety trade-offs. Treating these as the same target would blur endpoints and complicate attribution.

## 7 Conclusions and next steps

The CD160–HVEM axis is a context-dependent regulator of the immune set-point, distinct in its structural duality and in the epigenetic stability of the T-cell states it annotates. This review has adopted a three-part clinical geography—immuno-oncology, autoimmunity, transplantation—without dissolving the reciprocal paradigm that motivates the Research Topic.

State of play. While direct anti-CD160 candidates remain in advanced preclinical development, other components of the HVEM network are actively undergoing human clinical testing (**Table 3**; **Figure 3**). Tifcemalimab is an anti-BTLA antibody. Anti-HVEM reagents (Demerlé HVEM18-10; 4CB1) block both immunoglobulin ligands of the hub and are not interchangeable with each other or with anti-CD160. Direct anti-CD160 (CL1-R2, ELB01101, 22B12), CD160Ig and peptide A5 remain preclinical.

**Figure 3:**
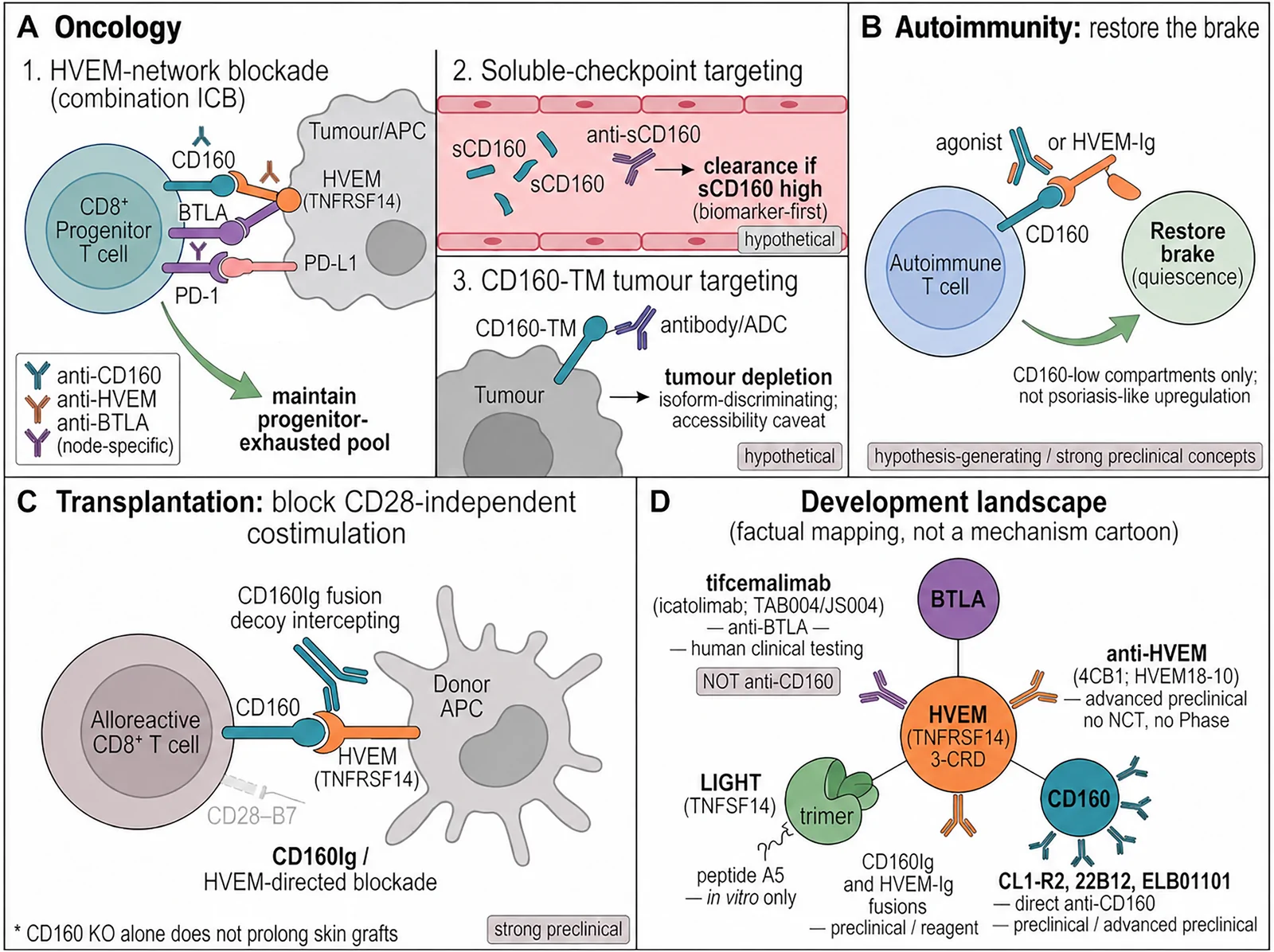
Precision strategies and clinical state of play in the HVEM network. Original schematic (redrawn from the authors’ AI artwork; not adapted from published diagrams). 2×2 layout. Map agents onto the node each engages; do not invent Phase numbers. **(A)** Oncology: 1. HVEM-network blockade (combination ICB): CD160 and BTLA on a progenitor CD8^+^ T cell both bind HVEM (TNFRSF14) on the tumour/APC; PD-1 on the T cell is paired with PD-L1 on the tumour. Node-specific antibodies (anti-CD160, anti-HVEM, anti-BTLA) sit on the HVEM-network contacts; a labelled anti-PD-1 is not drawn on the PD-1–PD-L1 pair. The intended readout is maintenance of the progenitor-exhausted pool. 2. Soluble-checkpoint targeting (biomarker-first; tagged hypothetical on the art): anti-sCD160 clearance if sCD160 is high. 3. CD160-TM tumour targeting (tagged hypothetical): isoform-discriminating antibody/ADC if tumours express CD160-TM. **(B)** Autoimmunity: restore the brake with agonist or HVEM-Ig on CD160, leading to quiescence; CD160-low compartments only, not psoriasis-like upregulation. This panel is a therapy cartoon, not a Healthy/Disease threshold diagram. **(C)** Transplantation: a CD160Ig fusion decoy intercepts CD160–HVEM costimulation on alloreactive CD8^+^ T cells versus donor APC (CD28–B7 faded). CD160 knockout alone does not prolong skin grafts. Sirolimus is not drawn as an HVEM-directed drug. **(D)** Development landscape (factual mapping, not a mechanism cartoon): tifcemalimab (icatolimab; TAB004/JS004) occupies the BTLA node — anti-BTLA, human clinical testing, explicitly not anti-CD160. Anti-HVEM (4CB1; HVEM18-10) occupies the HVEM (TNFRSF14) hub — advanced preclinical, no NCT, no Phase. Direct anti-CD160 (CL1-R2, 22B12, ELB01101) occupies the CD160 node — preclinical/advanced preclinical. LIGHT is labelled LIGHT (TNFSF14). Peptide A5 is *in vitro* only. CD160Ig and HVEM-Ig fusions are preclinical/reagent. Evidence levels: HVEM-network blockade and CD160Ig/agonism are strong preclinical; soluble-checkpoint (sCD160) targeting and CD160-TM tumour targeting are hypothesis-generating (as tagged on the art). Vascular normalisation is not a figure image panel (it is the **Figures 2A callout**). Panel **(D)** is a factual mapping of agents to nodes, not a causal claim.

Reciprocal take-home. Break pathological tolerance in cancer only with tools that do not erase the progenitor pool or NK IFN-γ; restore a lost inhibitory pairing in selected autoimmunity; block a CD28-independent CD8^+^ accelerator after transplantation. The same ectodomain cannot be asked to do all three jobs with one systemic antibody.

Three next steps. First, biomarker-qualified enrichment: isoform-resolved tissue profiling paired, where relevant, with circulating sCD160, plasma EV-CD160, and TLS density (47, 61, 64). Without alignment between mechanism and measurement, negative trials will remain uninterpretable. Second, isoform-discriminating reagents are a prerequisite deliverable, not an optional reagent catalogue (36, 52). Third, correlative CD160/HVEM profiling inside ongoing BTLA-directed human studies, including the tifcemalimab lymphoma (NCT04477772) and lung programmes (NCT05000684; NCT06095583 Phase 3 LS-SCLC consolidation) (75, 76).

Which agents look most promising (a working rank, not a prediction market). (i) Tifcemalimab/anti-BTLA is the most clinically mature node in this network and leaves the CD160 arm intact; it is the only agent we can currently place in human testing without fabricating a trial (73). (ii) Anti-HVEM hub blockade has the broadest mechanistic rationale and, in humanised mice, synergy with PD-L1 (38); 4CB1 remains preclinical/ex-vivo pending a registered trial (77). (iii) Isoform-specific anti-CD160-TM in TNBC is biologically the cleanest direct-CD160 idea and the earliest (52, 53). (iv) CD160Ig for transplantation is strong preclinical with no clinical path yet (56). (v) Peptide A5 remains *in vitro* (79). Local ocular anti-CD160 (ELB01101) is the most advanced direct anti-CD160 in animals, but it is an ophthalmology programme, not an oncology, autoimmunity or transplant trial (82).

What would change our mind. Clinical benefit of anti-HVEM with no change in CD160^+^ compartments would imply that CD160 is a passenger on this hub. Conversely, NK-toxic or progenitor-depleting signals from a pan-CD160 antibody would confirm the dual-function constraint in humans. A verified interventional trial of a direct anti-CD160 mAb would immediately rewrite the “preclinical only” sentences in Sections 3.4, 4.4 and 5.4.

Ultimately, the path to curing autoimmunity may well be paved by the mechanisms of tumour survival—provided those mechanisms are read with isoform, species and disease context attached.
